# Sub-Nanomolar Detection of Oligonucleotides Using Molecular Beacons Immobilized on Lightguiding Nanowires

**DOI:** 10.3390/nano14050453

**Published:** 2024-02-29

**Authors:** Therese B. Johansson, Rubina Davtyan, Julia Valderas-Gutiérrez, Adrian Gonzalez Rodriguez, Björn Agnarsson, Roberto Munita, Thoas Fioretos, Henrik Lilljebjörn, Heiner Linke, Fredrik Höök, Christelle N. Prinz

**Affiliations:** 1 Division of Solid State Physics, Lund University, 221 00 Lund, Sweden; therese.johansson@ftf.lth.se (T.B.J.); julia.valderas_gutierrez@ftf.lth.se (J.V.-G.);; 2 NanoLund, Lund University, 221 00 Lund, Sweden; 3 Division of Nano and Biophysics, Chalmers University of Technology, 412 96 Gothenburg, Sweden; 4 Division of Molecular Hematology, Department of Laboratory Medicine, Lund University, 221 00 Lund, Sweden; 5 Division of Clinical Genetics, Department of Laboratory Medicine, Lund University, 221 00 Lund, Sweden

**Keywords:** molecular beacons, nanowires, lightguiding, limit of detection, oligonucleotides

## Abstract

The detection of oligonucleotides is a central step in many biomedical investigations. The most commonly used methods for detecting oligonucleotides often require concentration and amplification before detection. Therefore, developing detection methods with a direct read-out would be beneficial. Although commonly used for the detection of amplified oligonucleotides, fluorescent molecular beacons have been proposed for such direct detection. However, the reported limits of detection using molecular beacons are relatively high, ranging from 100 nM to a few µM, primarily limited by the beacon fluorescence background. In this study, we enhanced the relative signal contrast between hybridized and non-hybridized states of the beacons by immobilizing them on lightguiding nanowires. Upon hybridization to a complementary oligonucleotide, the fluorescence from the surface-bound beacon becomes coupled in the lightguiding nanowire core and is re-emitted at the nanowire tip in a narrower cone of light compared with the standard 4π emission. Prior knowledge of the nanowire positions allows for the continuous monitoring of fluorescence signals from each nanowire, which effectively facilitates the discrimination of signals arising from hybridization events against background signals. This resulted in improved signal-to-background and signal-to-noise ratios, which allowed for the direct detection of oligonucleotides at a concentration as low as 0.1 nM.

## 1. Introduction

The detection of oligonucleotides, in the form of RNA, DNA, or cDNA, is central to many applications in biomedicine, such as assessing cell phenotype, diagnostics, and pathogen detection [1,2,3]. Molecular beacons (MBs) have been proposed for detecting oligonucleotides in the past due to their ability to fluoresce upon binding to complementary oligonucleotides, thereby enabling direct oligonucleotide detection without the requirement of labelling the target oligonucleotides [4]. MBs are hairpin-structured nucleic acid probes consisting of a single-stranded loop and a double-stranded stem. A fluorescent dye is attached to the end of one stand of the stem and a quencher to the end of the other strand. In the hairpin configuration, the MB fluorescence is quenched due to the close proximity of the fluorescent dye and the quencher. When complementary oligonucleotides are present, hybridization takes place, resulting in a loss of the hairpin configuration and an increased distance between the fluorophore and the quencher. Consequently, the MBs can fluoresce [4].

The detection of oligonucleotides using immobilized MBs on surfaces has been demonstrated in the past on various substrate materials such as planar glass, PMMA, and gold [5,6,7,8,9]. Gold surfaces were used as fluorescence-quenching materials, eliminating the need for incorporating a fluorescence quencher in the MB. One drawback of this approach is the relatively high background due to the MB’s non-specific opening and non-specific adsorption to surfaces, with a reported limit of detection (LOD) ranging from 100 nM to a few µM. Polymer brushes functionalized with MBs have been used as a passivating layer, enabling the detection of ≈1 nM of complementary oligonucleotides [10]. Another way to increase the MB signal is to amplify the fluorescence by enzymatic cleavage of multiple MBs, resulting in an LOD ≤ 100 fM. A limitation of this method is that the enzymes may cause non-specific digestion of the MBs, causing a false positive signal if an MB design is not thoroughly conducted [11]. In another approach, MBs encapsulated in hydrogels and spotted on surfaces in a microarray-like fashion also lead to better sensitivities, with an LOD of 0.1 nM. MBs immobilized on silica optical fibers were used to detect mRNA sequences amplified using PCR with an LOD of 1 nM [12].

More recently, nanostructures have been investigated to enhance the detection of MB hybridization. For instance, gold nanorod arrays functionalized with MBs induced local surface plasmon resonance-enhanced fluorescence of the hybridized MBs, enabling the detection of 0.1 nM of complementary oligonucleotides. Another example is photonic crystals. A shift in the photonic bandgap was measured in photonic crystals functionalized with MBs, due to changes in the refractive index resulting from MB hybridization to complementary oligonucleotides [13], with an LOD of 0.5 µM. In another study, the binding of fluorescently labeled miRNAs to complementary cDNA probes immobilized on a photonic crystal was detected with a 50-fold sensitivity improvement (down to 0.5 nM) compared with flat substrates [14,15]. The enhanced fluorescence was attributed to an excitation enhancement of the fluorophores close to the crystal surface and a directional beaming of emitted photons normal to the surface due to the presence of a grating on top of the crystal. While this method has not been tested on MBs per se, it is obvious that it could be applied to MB hybridization detection. However, a drawback of the method is that it requires careful alignment of the light incidence angle and polarization.

Semiconductor lightguiding nanowires (NWs) are another type of nanostructure providing excitation enhancement at their surface and directional emission of light emitted by fluorophores on, or close to, their surface [16,17,18]. NWs of high refractive index materials with an optimized diameter have been shown to enhance the signal from fluorescent molecules bound to their surfaces [17,19,20]. The light couples into the NW core and is emitted at the extremities in a preferred direction oriented along the NW axis, which results in an increased signal and reduced LOD [21]. Compared with photonic crystals, their fabrication is simpler and there is no requirement on the illumination scheme. Therefore, lightguiding nanowires are an interesting material for enhancing the fluorescence signal of MBs for the detection of oligonucleotides.

Here, we used lightguiding gallium phosphide NWs coated with SiO_2_ and functionalized with MBs to detect DNA oligonucleotides. As a proof of principle, we used MBs and complementary DNA oligonucleotides corresponding to a short segment of the ABL1 gene, which is associated with many processes in living cells, such as proliferation, cell death, cell differentiation, and cell migration [22]. The NW substrate was placed in a microfluidic device, and oligonucleotides were introduced at different concentrations while monitoring the MB fluorescence over time from thousands of individual NWs using epifluorescence. The data analysis was conducted by performing a linear fit of the intensity time-series data.

## 2. Materials and Methods

### 2.1. MB Design

The database of the National Center for Biotechnology Information [23] was used to identify the mRNA sequence for the ABL1 gene. As the target region, a section of the mRNA with a minimal degree of self-hybridization was chosen. The RNAfold web server [24] was used for this purpose. Once possible target regions were identified, the Basic Local Alignment Search Tool (BLAST) [25] was used to control their uniqueness for the ABL1 gene. An extra DNA sequence was attached to the MB to act as a linker for immobilizing the MB to the NW substrate. An effective MB should adopt a hairpin configuration. Therefore, the identified target regions, as well as the possible stem sequence and length, were tested by simulating the folding, self-hybridization, stability, and melting temperature using the Freiburg RNA tools [26] and Integrated DNA Technologies website [27]. The resulting optimal MB sequence was *TAT TTC TGA TGT CCA CCC CC*/TAO/**GCT GG**C ACG TTA ACA AAA GGA AGG GA**C CAG C**/ATTO647N/, where ATTO647N is the fluorophore, TAO is the corresponding quencher, the nucleotides in bold represent the stem, the sequence in italics represents the immobilization linker, and the remaining nucleotides form the loop targeting ABL1 mRNA.

Together with the molecular sequence above, the following oligonucleotide sequences were ordered from Integrated DNA Technologies (IDT):

Complementary oligonucleotide target: TCC CTT CCT TTT GTT AAC GTG.

Scrambled sequence oligonucleotide (negative control): AGT TCT CTC ATC GTT CCT TGT.

### 2.2. MB Functionality Control

The MBs and oligonucleotides were delivered from IDT in dried form. Before use, they were resuspended in TE-buffer (ThermoFisher Scientific Cat.no. J75793, Waltham, MA, USA) to a stock concentration of 100 µM. The functionality of the MBs in solution was tested using a fluorescence plate reader (CLARIOstar, BMG Labtech, Ortenberg, Germany). The MBs were diluted to a concentration of 250 nM in phosphate-buffered saline (PBS, Merck, Darmstadt, Germany, Cat.no. D8537) and added to a 96-well plate (Corning, New York, NY, USA, product no 3881) with a volume of 100 µL per well. Complementary oligonucleotide (250–1000 nM), scrambled oligonucleotide (500 nM), or DNase (0.251 U/µL) was then added to the MB solution. Due to transportation between the preparation site and the plate reader, the measurements were initiated at t = 20 min. The fluorescence in each well was measured for 10 h.

### 2.3. Nanowire Platform Fabrication and Characterization

GaP NW substrates were fabricated by Aligned Bio AB. The choice of GaP as the material was motivated by its indirect bandgap of 2.26 eV, resulting in negligible absorption of the light emitted by the MBs. GaP also has the benefit of being biocompatible, which is important for bioapplications [28,29].

A mask of 70 nm SiN was deposited on a 3” GaP(111)B wafer using plasma-enhanced chemical vapor deposition (PECVD, Microsys 200 ICP, MicroSystems GmbH, Berg, Germany), followed by two layers of resist, SF 3S and PAR1085S90, for pattern transfer. Displacement Talbot lithography (DTL, PhableR 100 DUV system, Würenlos, Switzerland) created a hexagonal nano pattern in the resist. By using reactive ion etching (RIE, Sirius T2 Plus table-top system, Trion technology, Clearwater, FL, USA), the pattern was transferred to the SiN mask. A layer of 60 nm of Au was deposited on the wafer. A lift-off procedure with Remover 1165 was then used to remove the resist, leaving only the Au seeds on the substrate at a density of 1.19 Au seeds/µm^2^. GaP NWs were grown from the gold seeds using metal–organic vapor phase epitaxy (MOVPE) in an Aixtron 200/4 reactor (Aixtron, Herzogenrath, Germany). The NWs were coated with a 10 nm layer of SiO_2_ using atomic layer deposition (ALD, Fiji, Cambridge Nanotech, Cambridge, MA, USA). See the Supplementary Information for more detail on the fabrication protocols. The NWs were characterized using scanning electron microscopy (SEM, LEO SEM, Zeiss, Jena, Germany). The final NW length was 2.43 µm ± 0.06 µm and the NW diameter was 142 nm ± 5 nm, which resulted in close-to-optimal light-collecting and light-guiding properties for the given fluorophore. From SEM images taken of the sample, the density of lightguiding NWs (vertical NWs) was estimated to be (1 ± 0.1)/µm^2^ (see S2 in the Supplementary Information for representative images of the NW arrays). The wafer was then diced into individual chips (2.5 × 2.5 mm^2^) using a DAD 3320 Dicer, DISCO, Munich, Germany. Prior to dicing, the wafer was coated with a photoresist layer of arbitrary thickness to protect the NWs from damage during dicing.

### 2.4. Device Design

**Figure 1:**
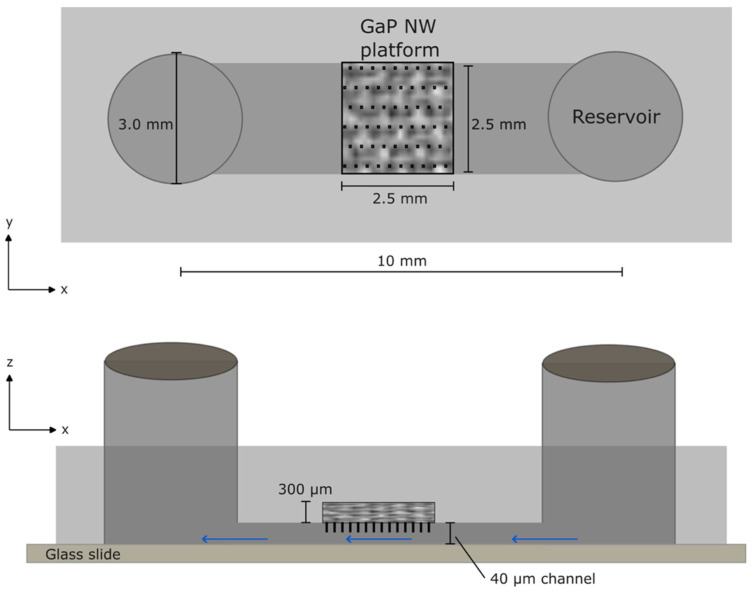
Schematics of the microfluidic device (top and side views). Reservoirs are connected to a microfluidic channel. Negative pressure is used to apply a flow in the channel (blue arrows).

The microfluidic devices used in the experiments were produced using soft lithography. A poly(dimethylsiloxane) (PDMS) cast was made by mixing a polymer base and curing agent (Sylgard^TM^ 184 Silicone Elastomer Kit, Midland, MI, USA) in a proportion of 10:1 and subsequently curing it in an oven at 80 °C for at least one hour. The resulting PDMS device cast consisted of a single channel of dimensions 10 mm×2.5 mm×40 µm with a hole of comparable dimensions to the ones of the nanowire substrate (2.5 mm×2.5 mm×275 µm) (Figure 1).

The PDMS cast was treated with air plasma (Plasmatic Systems, Inc., North Brunswick Township, NJ, USA) for 10 s and a glass slide (microscope coverslip, thickness 1.5) was treated with air plasma for 60 s. The nanowire substrate was attached in the designated hole made in the PDMS using silicone glue (Elastosil AO7, RTV-1 silicone rubber, Wacker Silicones, Wacker Chemie AG, Munich, Germany) and the PDMS cast was subsequently sealed to the coverslip. Holes with a diameter of 3 mm were punched in the device for the inlet and the outlet with a cutting tool. Reservoirs made of cut silicon tubing (inner diameter, 3 mm; outer diameter, 5 mm, 228-0707, Avantor, VWR, Radnor, PA, USA) with a volume of at least 300 µL were connected to the holes using silicone glue. A single-syringe infusion pump (Aladdin 1000, WPI, Sarasota, FL, USA) was connected to the outlet and delivered a negative-pressure-driven flow of 0.3 mL/hour in the system through capillary tubing.

### 2.5. Immobilization of MBs on the Nanowire Platform

**Figure 2:**
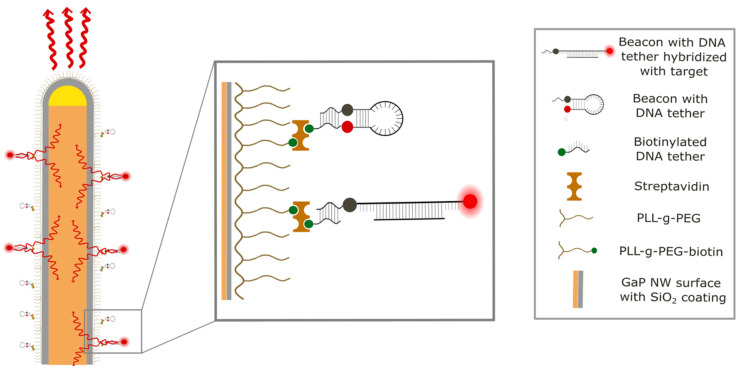
Schematic of the method. MBs are immobilized on the surface of lightguiding NWs. When hybridized with an oligonucleotide of complementary sequence, the MBs fluoresce. The emitted light then couples into the lightguiding NW core to be re-emitted at the tip in a narrow cone of light.

Layer-by-layer buildup enabled immobilization of MBs on the nanowire substrate (Figure 2). The different layers were added sequentially to the device through negative pressure, one at a time, with 60 min of incubation each, under constant flow (Aladdin 1000 one-syringe pump, WPI, 0.3 mL/h), and with abundant washing with PBS after each incubation (0.9 mL/h for 20 min).

First, the channel was flushed with a mixture of PLL-g-PEG and PLL(20 kDa)-graft[3.5]-PEG(2 kDa)/PEG(3.4 kDa)-biotin(50%) (PLL-g-PEG-biotin) (SuSos, premixed). Specifically, 3 µL of PLL-g-PEG-biotin at 10 mg/mL in PBS was mixed with 147 µL of PLL-g-PEG at 10 mg/mL in PBS and 150 µL of PBS. The PLL-g-PEG layer minimizes unspecific binding to the surface, while the biotinylated PEG is used to tether biotinylated DNA to the surface via streptavidin.

After rinsing, 225 µg/mL of streptavidin (Merck, Art.no. S4762) in PBS was added to the channel to build up the next layer, followed by 450 nM biotinylated DNA tether (Eurogentec, Seraing, Belgium). The DNA tether (C_8_H_18_O_5_—Biotin-5′-ATA-AAG-ACT-ACA-GGT-GGG-GG-3′) is complementary to the linker sequence of the MB and has a biotin with a tetraethylene glycol spacer arm attached to the 5′ end. Finally, 500 nM MBs with linker diluted in PBS were added to the channel.

### 2.6. Image Acquisition

Directly after immobilization of the MBs, the NWs were imaged using epifluorescence in an inverted fluorescence microscope (Eclipse Ti2, Nikon, Tokyo, Japan) with a 60 × 1.49 N.A. oil objective (Nikon, Tokyo, Japan), a Semrock FF01-698/70-Cy5 filter cube (Nikon, Japan), and a Sona -4BV11 sCMOS camera (Andor, Oxford instruments, Abingdon, UK). A 640 nm laser was used as an illumination source, at approximately 2 mW, corresponding to 2% of the maximum laser power. During the measurements, time-lapse images were acquired every 45 s with an exposure time set to 100 ms, with a total imaging time of 60 min per oligonucleotide target concentration. The same region on the NW substrate was imaged for all concentrations and used in the subsequent analysis.

### 2.7. Data Analysis

The process of data analysis was undertaken through a series of five distinct stages. A 1024 × 1024 pixel region (120 μm × 120 μm) was cropped in the center of the laser illumination area for all samples. Because the signal-to-noise ratio of each individual frame was too low to identify NWs, the detection of each NW location was achieved by applying a temporal averaging of each individual pixel using every image recorded for each concentration of target oligonucleotide. Due to the background fluorescence of MBs, time averaging enables the detection of NWs even without complementary oligonucleotides. Wavelet filtering and local gradient thresholding (described in [30]) were performed on raw, time-averaged images to accurately detect the nanowire positions.

Subsequently, the fluorescence intensity of individual NWs was monitored over time to quantify time-resolved changes in the fluorescence emission for each individual NW. Using linear regression analysis on the intensity time-series data, NWs were classified into three categories characterized by an intensity curve slope *k* with unit ΔI (A.U)/min: (i) NWs exhibiting an increase in fluorescence intensity (*k* > 0.5 ΔI (A.U)/min), (ii) NWs having a close to constant intensity (within the range of −0.5 ΔI (A.U)/min < *k* < 0.5 ΔI(A.U)/min), and (iii) NWs with decreasing fluorescence intensity (*k* < −0.5 ΔI (A.U)/min). For each oligonucleotide concentration *C*, the overall intensity change was plotted by adding the *k* values of all NWs exhibiting an increasing fluorescence intensity and subtracting the *k* values of all NWs exhibiting an increasing fluorescence when no oligonucleotide was present (*C* = 0), i.e. $\sum_{i=1}^{n(C)}k_{i}\left(C\right)-\sum_{i=1}^{n(0)}k_{i}\left(0\right)$.

## 3. Results 

### 3.1. MB Functionality in Solution

**Figure 3:**
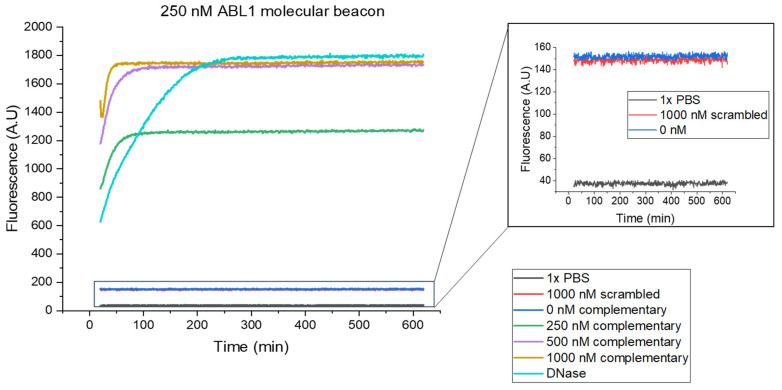
MB fluorescence signal in solution measured using a fluorescence plate reader. Fluorescence intensity over time of 250 nM MBs in PBS when adding different concentrations of complementary target oligonucleotides, scrambled sequence oligonucleotides, and DNase. Inset shows a close-up view of the fluorescence of PBS, 1000 nM scrambled oligonucleotide, and MBs only (0 nM). The measurements were initiated at 20 min after the introduction of the oligonucleotides in the MB solution.

The MB functionality and specificity in bulk were evaluated using a multi-well fluorescence plate reader. MBs at a concentration of 250 nM in PBS were mixed with different concentrations of complementary target oligonucleotide, and the resulting MB fluorescence was measured for 10 h using a fluorescence plate reader (Figure 3). The background fluorescence signal of the MBs without oligonucleotide (Figure 3 inset, blue curve) was stable over the duration of the measurement at a value almost four times higher than that of PBS (Figure 3 inset, black curve). The MB background is due to insufficient quenching and non-specific opening with MBs varying constantly between the open and closed state at a very fast rate (≈µs) [5].

In the presence of the complementary target oligonucleotide, the MB fluorescence signal increases due to hybridization. An increased concentration of the complementary target results in an increased fluorescence signal until saturation occurs. Here, for the given MB concentration of 250 nM, saturation had already been achieved for the complementary oligonucleotide concentration of 500 nM (Figure 3, purple curve) since no additional binding was observed at higher oligonucleotide concentrations (Figure 3, brown curve), suggesting an apparent dissociation equilibrium constant *K_d_* of around 35 nM (see Supplementary Material S3). Adding DNase to the beacon solution led to the total degradation of the MBs and therefore the highest fluorescence intensity (Figure 3, cyan curve). No increase in fluorescence was observed when a scrambled oligonucleotide was used (Figure 3 inset, red curve). Overall, the fluorescence plate reader measurements indicate a good specificity of the MBs and low background fluorescence.

### 3.2. Detection of Complementary Oligonucleotide on the NW Platform

**Figure 4:**
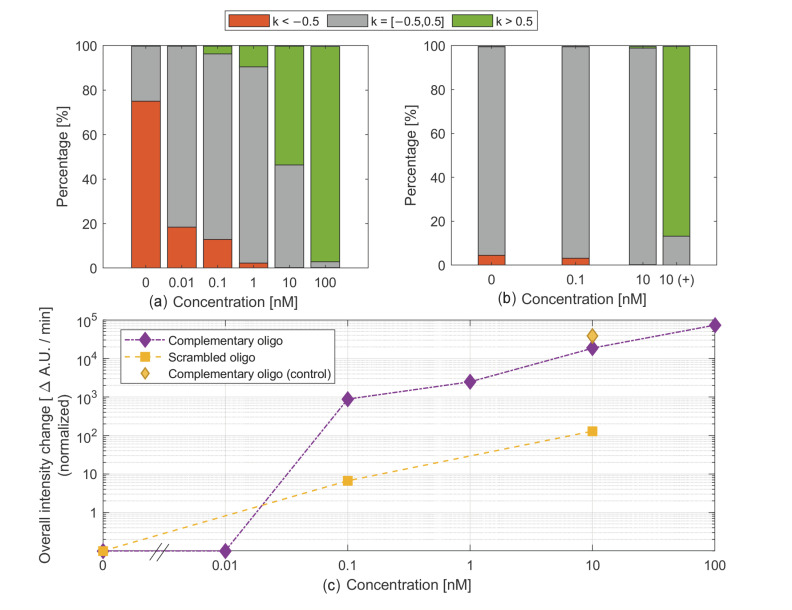
Analysis of the fluorescence of MBs immobilized on GaP NWs when target complementary oligonucleotides (**a**,**c**) and scrambled oligonucleotide sequences (**b**,**c**) are introduced into the device. (**a**,**b**): Percentage of NWs exhibiting a fluorescence intensity increase over time (green, line coefficient *k* > 0.5 A.U./min), a decrease over time (red, *k* < −0.5 A.U./min ), and NWs with a stationary fluorescence intensity (gray, −0.5 A.U./min < *k* < 0.5 A.U./min). (**c**): Sum of the line coefficients *k* for the NWs with an increasing fluorescence signal only.

The NW platform was used to assess the limit of detection of target complementary oligonucleotides using MBs immobilized on the NW substrate. In these measurements, the MB fluorescence was measured over time on each individual NW using fluorescence microscopy, and a linear regression analysis of the intensity was performed (average line coefficient, see the Section 2.). The nanowire emission was classified into three categories, defined by the average line coefficient *k* characterizing the temporal evolution of the fluorescence emission of individual nanowires: decreasing (*k* < −0.5 A.U./min), unchanged (−0.5 A.U/min < *k* < 0.5 A.U./min), and increasing (*k* > 0.5 A.U./min). At a 0 nM complementary oligonucleotide concentration, >70% of the NWs exhibited a fluorescence intensity decrease over time (the proportion of NWs colored in red in Figure 4a), which was attributed to bleaching. For all target concentrations, there was a fraction of NWs (in gray in Figure 4a) with stationary fluorescence. Upon the addition of an increasing target concentration, the percentage of NWs with increasing and decreasing fluorescence emissions increased and decreased, respectively. While a reduction in the percentage of NWs that exhibit a decrease in fluorescence emissions, as observed at 0.01 nM, may be due to target binding to beacons that counteract bleaching, the existence of nanowires with increasing fluorescence emissions at 0.1 nM can with high certainty be attributed to target binding and defines the limit of detection of the assay.

This is further supported by plotting the normalized sum of the line coefficients *k* for NWs with an increasing fluorescence signal only, which increases from a 0.1 nM target concentration and up (Figure 4c, purple diamonds). In order to determine the specificity of the assay, control experiments were carried out using scrambled oligonucleotides instead of the complementary oligonucleotide targets. For all concentrations of scrambled oligonucleotides, the percentage of NWs with a constant intensity over time was close to 100% (gray in Figure 4b), indicating that there was no hybridization when scrambled oligonucleotides were added to the MBs. The normalized sum of the line coefficient *k* of all NWs with an increasing fluorescence signal remained low, at least two orders of magnitude lower than when complementary oligonucleotides of the same concentration were used (Figure 4c, orange squares). Note that it is not equal to zero in this case, which is due to ≈30 NWs (out of ≈115,000 NWs) exhibiting a mildly increasing intensity, which we speculate can be attributed to inhomogeneous MB coverage and possibly local laser-induced heating of the MBs. As an additional control, 10 nM of complementary oligonucleotides was added to the channel at the end of the measurements performed with scrambled oligonucleotides. The percentage of NWs with an increasing fluorescence signal went up to almost 80% (Figure 4b 10_(+)_) and the sum of the line coefficients *k* for the NWs with an increasing fluorescence signal increased by a factor of ≈100 (Figure 4c, orange diamond) compared with scrambled oligonucleotides to a value that is consistent with previous measurements using the same complementary oligonucleotide concentration.

The decrease in fluorescence observed in some NWs is proposed to be due to bleaching. The percentage of NWs with a decreasing intensity at a 0 nM complementary oligonucleotide concentration is much larger in Figure 4a than for the control in Figure 4b. We speculate that this difference is mostly due to variations between NW samples, such as potential contamination and the tilt of the platform. For instance, there may be organic residues on some NWs due to the use of resist to protect them during dicing. This could possibly lead to different MB coverages between NWs. Moreover, a small tilt of the NW platform in a direction perpendicular to the flow direction can easily happen due to the mode of fixation of the platform in the channel and may also result in uneven MB surface concentrations since the reagent concentrations will be slightly uneven on the sample. Variations in MB surface coverage between NWs can affect their intensity change in the absence of target oligonucleotides. For instance, it is possible that the MB background signal on some NWs with a lower degree of MB coverage will not be detected with sufficient sensitivity to be able to measure the decrease in fluorescence intensity (attributed to bleaching) that we can detect on NWs with a hypothetically higher degree of MB surface coverage. However, more experiments would be necessary to determine the origin of the sample-to-sample variation with certainty.

## 4. Discussion

A unique advantage of our method is that it enables sub-nanomolar oligonucleotide detection without the need for amplification, which surpasses many other methods involving MB fluorescence. The key feature of the method is the use of the lightguiding properties of the NWs to effectively collect and direct the fluorescence signal emitted by the hybridized MBs without the need to amplify the target concentration or fluorescence signal. Moreover, with lightguiding NWs, since the light is collected at the tip of the NWs, there is no requirement to use a transparent fluid during the measurements, which opens up the possibility of doing measurements in complex fluids. Previous studies have also demonstrated that the hybridization efficiency is facilitated by the curvature arising from the nanostructured surface compared with when MBs are immobilized on a flat glass surface [9], which could be an additional benefit of our assay.

Some studies, however, have reported an LOD in the fM range. Luan et al. [31] reported an LOD of 0.5 fM with MBs immobilized on a thin layer of gold and using surface plasmon resonance fluorescence enhancement for the detection of complementary target DNA. Using paper-based lateral flow strips, Moon et al. reported an LOD down to the sub-femtomolar level for MB detection of a target nucleic acid [32].

Our method could possibly be improved by using a higher degree of MB coverage. For instance, Cederquist et al. [9] immobilized MBs on gold NWs in order to investigate how the hybridization efficiency was affected by the MB coverage. They showed that the hybridization efficiency increases as the MB surface density increases for coverages between 0.3 × 10^12^ MB/cm^2^ and 1.2 × 10^12^ MB/cm^2^. At higher coverages, there was a drastic drop in the hybridization efficiency from 90% to approximately 20%. In our experiments, the MB coverage was estimated to be 3.5 × 10^10^ MB/cm^2^ by assuming a PLL binding area on the surface of 10 nm × 10 nm [33]. Our estimated MB coverage is lower than the ones used by Cederquist et al. This suggests that, in our case, the hybridization efficiency can possibly be improved by increasing the MB coverage due to the reported effects of MB density on hybridization.

Due to practical issues related to the synthesis of MBs, the quencher had to be placed next to the DNA linker. Swapping the positions of the fluorophore and quencher in the MB sequence would lead to the fluorophore being ≈10 nm closer to the NW surface after hybridization and thereby result in a stronger coupling of the MB fluorescence in the NW core [16]. NWs could also be modified to enhance the coupling of light. For instance, in the future, advanced light manipulation in NWs may be possible by tuning the NW index of refraction axially at the nanoscale [34]. Moreover, although using a core–shell geometry may lead to less effective in-coupling of emissions and out-coupling of excitation due to the increased confinement, it could offer an additional degree of freedom in the design and optimization of lightguiding nanowires [35].

In the future, the costs of the present NW platform could be reduced by synthesizing the NWs using aerotaxy instead of epitaxy. Aerotaxy is an aerosol-based NW synthesis material that results in faster growth without the use of a substrate [36]. Aerotaxy-grown lightguiding NWs have been shown to have similar properties to the ones grown using MOVPE [30]. Furthermore, the use of other materials than GaP, such as silicon, might further reduce the costs.

A drawback of the present method is the relatively large volume (300 µL) of fluid needed in the device, which can be challenging when aiming at detecting rare targets. Adjusting the device design, including the channel dimensions, could enable smaller volumes to be used. Moreover, achieving the automation of sample handling and image acquisition during surface functionalization and subsequent measurements would result in less variability between experiments compared with when performing these steps manually.

## 5. Conclusions

Fluorescent MBs were immobilized on lightguiding NWs and used to detect oligonucleotides of complementary sequences using fluorescence microscopy. The fluorescence intensity was analyzed by determining the average line coefficient *k* characterizing the temporal evolution of the fluorescence emission of individual NWs. For each oligonucleotide concentration tested, we calculated the normalized sum of the line coefficient *k* for the NWs exhibiting an increasing line coefficient only. Using this method, we detected complementary oligonucleotides at a concentration as low as 0.1 nM, as shown by a normalized sum of the line coefficient *k* two orders of magnitude larger than the one corresponding to non-complementary oligonucleotides present at the same concentration. The benefit of the method is that it allows for direct measurements without the need to amplify the fluorescence signal. Possible improvements to achieve the detection of low-abundance oligonucleotides are the further miniaturization of the platform and the design of a new MB with a fluorophore in closer proximity to the NW surface upon MB hybridization for optimized signal quality.

## Data Availability

The data presented in this study are available on request from the corresponding author.

## Supplementary Materials

The following supporting information can be downloaded at: https://www.mdpi.com/article/10.3390/nano14050453/s1, S1: NW platform fabrication. S2: Scanning electron microscopy imaging of the NW substrate. S3: Estimation of the dissociation constant Kd from plate reader measurements
