# Supplementary material for: Sub-Nanomolar Detection of Oligonucleotides Using Molecular Beacons Immobilized on Lightguiding Nanowires
Source: Nanomaterials (Basel). 2024 Feb 29;14(5):453. doi: 10.3390/nano14050453 (PMC10934797; doi:10.3390/nano14050453)
Supplement: Supplementary file 1 [file nanomaterials-14-00453-s001.zip › nanomaterials-2867804-supplementary.pdf]

SUPPLEMENTARY INFORMATION

Sub-Nanomolar Detection of Oligonucleotides Using Molecular Beacons Immobilized On Lightguiding Nanowires

Therese B. Johansson [1,2,†], Rubina Davtyan [1,2,†], Julia Valderas-Gutiérrez, [1,2], Adrian Gonzalez Rodriguez [3,‡], Björn Agnarsson [3], Roberto Munita [4,§], Thoas Fioretos [5], Henrik Lilljebjörn [5], Heiner Linke [1,2], Fredrik Höök [2,3,*] and Christelle N. Prinz [1,2,*]

[1] Division of Solid State Physics, Lund University, 221 00 Lund, Sweden

[2] NanoLund, Lund University, 221 00 Lund, Sweden

[3] Division of Nano and Biophysics, Chalmers University of Technology, 412 96 Gothenburg, Sweden

[4] Division of Molecular Hematology, Department of Laboratory Medicine, Lund University, 221 00 Lund, Sweden

[5] Division of Clinical Genetics, Department of Laboratory Medicine, Lund University, 221 00 Lund, Sweden

[*] Correspondence: fredrik.hook@chalmers.se (F.H.); christelle.prinz@ftf.lth.se (C.N.P.)

[†] These authors contributed equally to this work.

[‡] Current address: Bosch, 1023 Budapest, Hungary.

[§] Current address: Advanced Center for Chronic Diseases (ACCDiS), Department of Biochemistry and Mo-lecular Biology, Faculty of Chemical and Pharmaceutical Sciences, Universidad de Chile, 8380494 Santiago, Chile.

S1: NW platform fabrication.

A layer of 70 nm thick SiN mask was deposited on a 3”(111)B wafer using PECVD (Micro Systems 200 ICP PE-CVD). The chamber heater was set at 250°C, the RF power for the Ar plasma was set at 300 W. The precursor gas flows were 50 SCCM for Ar, 10 SCCM for silane, and 11.5 SCCM for ammonia.

The wafer was then spin-coated with the bottom anti-reflection coating SF 3S, at 2000 RPM for 45 s, baked at 200° C for 10 min on a hotplate and cooled down before the deep-UV resist PAR1085S90 was spin coated on the wafer at 4500 RPM, and bake at 90°C for 1 min.

The wafer was then exposed using the Displacement Talbot Lithography system PhableR 100 DUV system to transfer an hexagonal pattern in the resist. The instrument settings were as follows: Wavelength of light: 193 nm; frequency:100 Hz; dose: 3-3.5 mJ/cm^2: separation: 80 mm; Talbot distance: 8 mm; pulse energy: 1.5 mJ, Power: 38 mW/cm^2. The wafer was then baked on a hotplate

at 100°C for 50 s. After cooling down, the wafer was developed in MF24A for 60 s before being transferred to a deionized water bath and further rinsed under gentle deionized water flow.

To transfer the hexagonal pattern into the SiN layer, RIE (Sirus T2 Plus table-top RIE system) was performed at 500 mTorr, RF power 75W with 5 SCCM of CF_4, 5 SCCM of CHF_3, and 50 SCCM of O_2.

Subsequently, 60 nm of 24K gold, 99.95 % purity were deposited on the wafer using evaporation (Temescal E-Beam evaporator) and lift-off was performed in 1165 remover.

The NW growth was performed in an Aixtron 200/4 MOVPE reactor at 480° with trimethylgallium (molar fraction 9.83 10^{-5} a.u.), Phosphine (molar fraction 2.31 10^{-2} a.u.) and HCl (molar fraction 1.85 10^{-4} a.u.). Finally, the NWs were coated with a 10 nm-layer of SiO2 using atomic layer deposition (ALD, Fiji). The wafer with NWs was subsequently coated with a photoresist layer of arbitrary thickness and diced into individual chips, 2.5×2.5 mm^2, using a Disco DAD 3320 Dicer. After dicing, the chips were washed in acetone for 30 min and in isopropanol for 30 min and dried using a nitrogen gun. The chips were then treated with UV-ozone for 90 min at 90°C (UVOH 150, FHR Anlagenbau GmbH) and stored in a glovebox protected from air and light until use.

S2: Scanning electron microscopy imaging of the NW substrate.

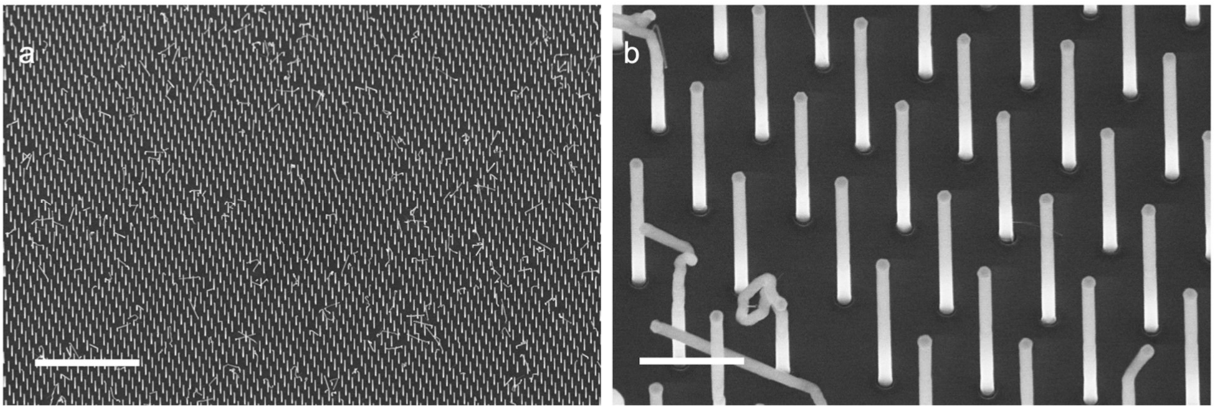

Figure S1: Representative scanning electron microscopy images of the GaP NWs. InLens detector, stage tilt 30°, accelerating voltage 15 kV, working distance 11 mm. Scale bars 10 µm (a) and 1 µm (b). After eliminating all NWs deviating from a rod-like morphology oriented perpendicular to the substrate, the density of light guiding NWs on the substrate was estimated to be 1±0.1/μm^2. Note that the kinked NWs do not disturb the measurements, as they are not detected in fluorescence microscopy.

S3: Estimation of the dissociation constant K_d from plate reader measurements (Fig. 3).

The dissociation constant is defined by:

$$K_d = \frac{[MB]\,[Oligo]}{[MB - Oligo]} = \frac{([MB]_0 - [MB - Oligo])\,([Oligo]_0 - [MB - Oligo])}{[MB - Oligo]}$$

where:

$[MB]$ is the concentration of unhybridized MBs

$[MB]_0$ is the initial concentration of MBs = 250 nM

$[Oligo]$ is the concentration of unhybridized oligonucleotides

$[Oligo]_0$ is the concentration of added oligonucleotides

$[MB - Oligo]$ is the concentration of hybridized MBs

The intensity of the hybridized MBs in the plate reader can be written as:

$I = a[MB - Oligo] + b$,

where a and b are constant.

From the intensities displayed for different oligonucleotide concentrations displayed in Figure 3:

b is the background = 150 A.U.

In the presence of DNAse, all MBs are degraded and the intensity I = 1750 is the same as in the case $[MB - Oligo]$ = $[MB]_0$= 250 nM, therefore $a = \frac{1600}{250} = 6{,}4$.

From the case where $[Oligo]_0 = 250nM$, where I = 1250, one can deduct that at this concentration of oligonucleotide: $[MB - Oligo] = \frac{1100}{6.4} nM \approx 172\, nM$

Therefore

$$K_d = \frac{([MB]_0 - [MB - Oligo])\,([Oligo]_0 - [MB - Oligo])}{[MB - Oligo]} = \frac{(250 - 172)^2}{172} nM \approx 35\, nM$$
